# Diagnostic and Immunosuppressive Potential of Elevated Mir-424 Levels in Circulating Immune Cells of Ischemic Stroke Patients

**DOI:** 10.14336/AD.2017.0602

**Published:** 2018-04-01

**Authors:** Guangwen Li, Qingfeng Ma, Rongliang Wang, Zhibin Fan, Zhen Tao, Ping Liu, Haiping Zhao, Yumin Luo

**Affiliations:** ^1^Cerebrovascular Diseases Research Institute and Department of Neurology, Xuanwu Hospital of Capital Medical University, Beijing, China; ^2^Beijing Geriatric Medical Research Center and Beijing Key Laboratory of Translational Medicine for Cerebrovascular Diseases, Beijing, China; ^3^Beijing Institute for Brain Disorders, Beijing, China

**Keywords:** stroke, miR-424, lymphocytes, neutrophils, CDK6, ischemia

## Abstract

Our previous study demonstrated that microRNA-424 (miR-424) protected against experimental stroke through inhibition of microglial proliferation and activation by targeting cell cycle proteins. The purpose of this study was to further explore the clinical significance of miR-424 in peripheral immune cells of patients with acute ischemic stroke (AIS). Blood samples were collected from 40 patients within 6 hours of symptom onset and 27 control subjects. MiR-424 levels in lymphocytes, neutrophils and plasma were determined by quantitative realtime-PCR. The diagnostic sensitivity and specificity of miR-424 for stroke was evaluated by receiver operator characteristic (ROC) curve. The correlation between miR-424 levels and clinical data was analyzed using Pearson’s correlation test. Plasma levels of inflammatory mediators (TNF-α, IL-10) and neurotrophic factor (IGF-1) were detected by ELISA. Notably, miR-424 expression levels in lymphocytes and neutrophils increased after stroke, suggestive of its diagnostic value in ischemic stroke. MiR-424 levels in neutrophils were negatively correlated with infarct volume. Lymphocytic miR-424 levels were negatively correlated with the number of lymphocytes and the expression of cyclin-dependent kinase CDK6. Moreover, plasma TNF-α and IGF-1 levels increased and decreased, respectively, in stroke patients, and miR-424 levels in lymphocytes and neutrophils were both inversely correlated with plasma TNF-α, IL-10, or IGF-1 levels. In summary, miR-424 levels in peripheral immune cells has diagnostic potential for ischemic stroke, and might affect the severity of acute stroke by depressing the peripheral inflammatory response through CDK6-dependent pathway in lymphocytes or CDK6-independent pathway neutrophils.

An increasing number of randomized trials involving patients with acute ischemic stroke (AIS) have shown a clinical benefit for intravenous thrombolysis within 4.5 hours, [1] or endovascular therapy within 8 hours after symptom onset [2,3]. However, only 2-3% of AIS patients receive intravenous thrombolysis and 4-14% are eligible for endovascular therapy due to a short therapeutic window [4,5]. Therefore, early diagnosis and reperfusion therapy in AIS patients is essential to reducing mortality and morbidity. To date, computed tomography (CT) and magnetic resonance imaging (MRI) are often used in the diagnosis of AIS, but neither provide enough diagnostic and prognostic information in stroke management. There is a lack of specific and sensitive biomarkers to distinguish AIS patients. An ideal diagnostic biomarker should provide insight into associated risk factors, and help predict prognosis to guide medical management [6].

The use of plasma biomarkers is thought to be the most valuable adjunct to physical examination. MicroRNAs (miRs) are endogenous short non-coding RNAs that repress gene expression post-transcriptionally [7]. MiRs exist in all human cells, and play an essential role in many physiological and pathological processes [7, 8]. MiRs levels in the core of brain infarction and peri-infarction area can change rapidly after stroke in a time-dependent manner, [9] and in some conditions, correlates with the expression of miRs levels in the blood of ischemic mice [10, 11]. Therefore, miRs in circulating blood can be used to diagnose or guide management of AIS.

The immune response after stroke plays a major role in the ischemic brain. The peripheral and central immune cells can significantly predict and affect the clinical outcome of stroke. Therefore, they constitute a potential target for treatment and prevention of stroke. Our previous study showed that plasma miR-424 level in AIS patients significantly decreased at 24 hours after symptom onset [12]. Further experimental research showed miR-424 levels in the brain remarkedly decreased at 24 hours post-ischemia and played a neuroprotective function against the ischemic brain through inhibition of microglial proliferation and activation by targeting cell cycle proteins including cyclin D1, CDK6, and CDC25A [12, 13]. Given its immunoregulatory function in the central nervous system, we investigated in this study the diagnostic and immunoregulatory potential of miR-424 expression in circulating immune cells during the acute stage of ischemic stroke by detecting the alteration of miR-424 levels in circulating blood (specifically, in lymphocytes, neutrophils, and plasma), as well as analyzing the correlation between miR-424 levels and the peripheral inflammatory response in ischemic stroke.

## MATERIALS AND METHODS

### Clinical Patient Selection and Patient Characteristics

This project was approved by the Committee of Institutional Review Board of Capital Medical University, Beijing, China. Written and oral informed consent was obtained from patients or legal representatives according to the Helsinki Declaration, which was adhered to during the studies. This prospective study analyzed 40 ischemic stroke patients in the emergency department or neurology ward of Xuanwu Hospital from March to December 2015. 27 control volunteers without any focal neurological deficits and history of CNS disease were recruited from medical examination center of Xuanwu Hospital, Capital Medical University, China. The control group as matched in age and sex to the acute stroke group. The diagnosis of ischemic stroke was made by neurologists based on the patient’s medical history, physical examination and radiologic findings on admission in accordance with guidelines formulated in 2014 [14]. The inclusion criteria were as follows: 1) first ischemic stroke and admission within 6 hours of symptom onset; 2) National Institute of Health Stroke Scale (NIHSS) <25 points; 3) sudden occurrence of focal neurological deficits with exclusion of hemorrhage on CT; 4) adequate access to patient information. The exclusion criteria included: 1) recurrent stroke; 2) hematologic diseases, malignant tumors, renal or liver failure; 3) history of mental disorders, severe dementia or coronary artery disease; 4) other diseases affecting the hemogram. All patients underwent a general medical evaluation and standard neurological and, including an assessment using NIHSS score at admission and at 7 days after thrombolytic therapy.

### Evolution of cerebral infarct volume assessed by diffusion-weighted magnetic resonance imaging

The location and volume of cerebral infarction were determined by MRI within 72 hours of stroke onset on a Magnetom Verio syngo 3.0T (Siemens, Germany) (n=24). Images were transmitted to picture archiving and communication systems (PACS). The infarct location was supplied by the middle cerebral artery. A blinded radiologist used diagnostic workstations with unisight system to interpret and measure the infarct volume. The total infarct volume was calculated by multiplying the infarct size on the diffusion-weighted imaging (DWI) sequence by the thickness. All the imaging data was standardized by Cross-Sectional Area Intracranial [15]. The formula of the volume standardization of cerebral infarction is as follows: infarct volume × mean of intracranial cross-sectional area/intracranial cross-sectional area of patient.

### Blood Preparation and miRNA Extraction

Blood samples from stroke patients were collected within 6 hours of stroke symptom onset. Blood sample of patients (4 ml×2) was collected into tubes containing ethylenediaminetetraacetic acid (EDTA) by venipuncture prior to administration of any therapies. The samples were processed as accordingly: first, the sample was centrifuged at 200 g for 10 minutes at 4? immediately for plasma extraction, and then the plasma was fractionated into vials with RNase/DNase-free and stored at -80? for further testing. Blood cells were then diluted with 8 ml NS and added to the surface of the lymphocyte separation medium (Tian Jin Hao Yang Biological Manufacture Co., Ltd., China) slowly in two 15 ml centrifuge tubes. After the tubes were centrifuged at 400 g for 20 minutes at 20?, the lymphocytes were separated and saved. Lastly, erythrocytes were dissociated with erythrocyte lysing solution and the remaining neutrophils were saved. Total RNA in neutrophils and lymphocytes was extracted using TRIzol method according to the manufacturer’s protocol and stored at -80? with RNase/DNase-free tubes for further testing.

### Quantitative Real-time Polymerase Chain Reaction (qRT-PCR)

Total RNA in plasma was extracted using TRIzol LS reagent (Invitrogen, Carlsbad, CA, USA) and RNA in neutrophils and lymphocytes was extracted using TRIzol reagent (Invitrogen, Carlsbad, CA, USA) according to the manufacturer’s protocol. Total RNA (300 ng) was reverse transcripted to cDNA using the Superscript III reverse transcriptase kit (Invitrogen, Carlsbad, CA, USA). After the first-strand cDNA was synthesized from total RNA, the amplification was carried out using a thermal cycle program consisting of 16 ? for 30 minutes, 42 ? for 40 minutes and 85 ? for 5 minutes, and cDNA was stored at -20 ? for further processing. The Gene Amp PCR System 9700 (Applied Biosystems) was used for the TaqMan-based real-time reverse transcription polymerase chain reaction (RT-PCR) assays. The primers and probes of the miRNA-424-5p and U6 endogenous controls for miRNA assays were purchased from Shanghai Bioligo Technology Co. Ltd: the PCR primers for miR-424 were 5’-GGC AGC AGC AAT TCA TG-3’ and 5’-CAG TGC GTG TCG TGG AGT-3’; the PCR primers for U6:5’-GCT TCG GCA GCA CAT ATA CTA AAA T-3’ and 5’-CGC TTC ACG AAT TTG CGT GTC AT-3’. Real-time PCR was performed according to the manufacturer’s protocols. Relative gene expression was normalized and expressed as fold-change relative to that of U6 and calculated via a 2^-△△CT^ method.

### Clinical Parameters and Measurements

Two 4 ml blood samples were collected into a vacuum tube containing 2.0 mg/ml EDTA-2K by venipuncture from all stroke patients at time of admission and preserved at 37 ? for routine laboratory assays immediately. Tumour necrosis factor-alpha (TNF-α), insulin-like growth factor 1 (IGF1), and interleukin-10 (IL-10) levels in plasma were measured with enzyme-linked immunosorbent assay (ELISA) kits according to the instructions and all the kits were purchased from Neobioscience Technology Company (China).

### Statistical analysis

Values in the study are presented as means ± SEM. Independent samples t-test was used for two-group comparisons. Qualitative data was presented as counts and percentages, and was compared with Fisher Exact test. One way analysis of variance (ANOVA) was used to compare several quantitative variables. Receiver operator characteristic (ROC) curve analysis was performed to calculate the predictive power of the sensitivity and specificity for the diagnosis of ischemic stroke. The overall diagnostic accuracy of the models was assessed using the area under the receiver operating characteristic curve (AUC). The correlation between two variables was analyzed using the Pearson’s correlation test. Statistical analysis was performed using the SPSS Version 17.0 software (SPSS Inc., Chicago, IL, USA). The exact *p* value was calculated and a probability of *p*<0.05 was considered to be statistically significant.

**Table 1:** Baseline characteristics of AIS patients and controls.

Characteristics	AIS patients (n=40)	Controls (n=27)	*p* value
Age (years)	64.6±10.3	62.2±12.7	0.374
Gender (M/F)	25/15	16/11	0.789
Hypertension, n (%)	14 (35.00)	8 (29.62)	0.646
Diabetes, n (%)	8 (20.00)	4 (14.81)	0.578
Hyperlipidemia, n (%)	7 (17.50)	4 (14.81)	0.771

**Figure 1.**
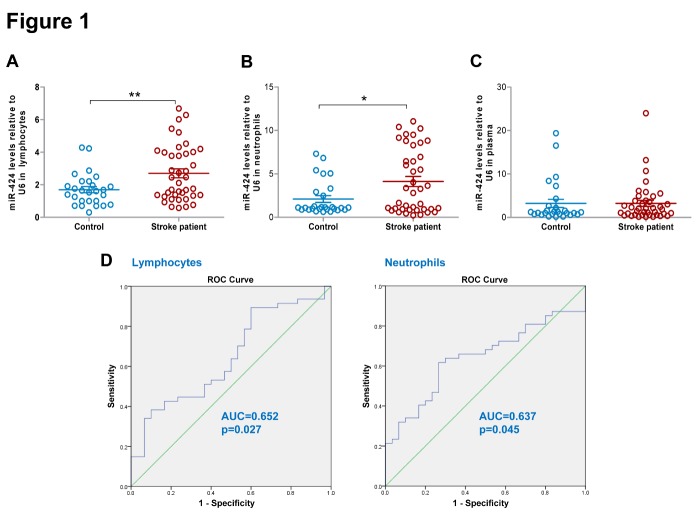
Changes of circulating miR-424 levels and its diagnostic value in hyperacute ischemic stroke. Quantitative real-time PCR analysis of the expressions of miR-424 in (A) lymphocytes; (B) neutrophils; and (C) plasma from AIS patients within 6 hours of symptom onset (n=40) and control group (n=27). Data represent mean ± SEM. * *p*<0.05 compared to control; **p<0.01 compared to control. (D) The diagnostic sensitivity and specificity of miR-424 levels in lymphocytes for AIS patients (n=40) was evaluated by ROC curve. ROC= receiver operator characteristic.

## RESULTS

### Baseline characteristics

A total of 40 ischemic stroke and 27 healthy volunteers were enrolled in this study. There was no statistically significant difference in age, sex, or risk factors, including a history of diabetes, hypertension or hyper-cholesterolemia (Table 1).

### Diagnostic value of miR-424 in AIS patients within 6 hours

Lymphocytes and neutrophils are the most abundant white blood cells, constituting more than 90% of the circulating leukocytes [16]. We first screened the expression of miR-424 in lymphocytes and neutrophils of all stroke patients and healthy control subjects using qRT-PCR. MiR-424 levels in lymphocytes and neutrophils both significantly increased within 6 hours of stroke onset (Fig. 1A-B, *p*<0.05). However, miR-424 levels in plasma did not change significantly (Fig. 1C).

**Table 2:** Diagnosis of stroke using miR-424 levels from AIS patients within 6 hours.

MiRs	Cells	AUC	95%CI	*p* value	Cut-off point	Sensitivity	Specificity
MiR-424	Lymphocytes	0.652	0.527-0.777	0.027	2.696	0.600	0.900
	Neutrophils	0.637	0.512-0.762	0.045	1.300	0.600	0.733

**Figure 2.**
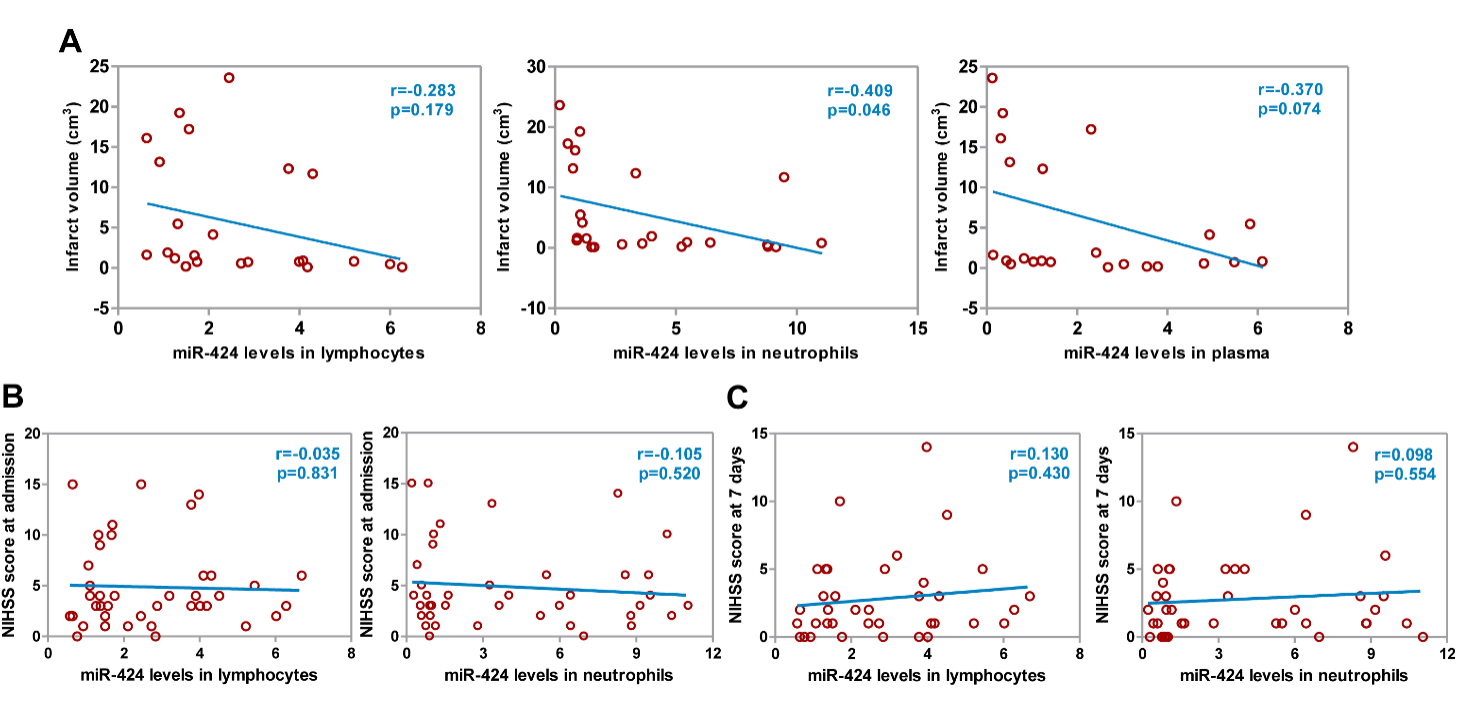
Correlations between miR-424 levels in circulating blood and cerebral infarct volume or NIHSS score. (A) Correlation between miR-424 levels in lymphocytes, neutrophils, and plasma and cerebral infarct volume within 6 hours of symptom onset in 24 AIS patients at admission; (B) Correlation between miR-424 levels in lymphocytes and neutrophils within 6 hours and NIHSS score in 40 AIS patients at admission; (C) Correlation between miR-424 levels in lymphocytes and neutrophils within 6 hours and NIHSS score in 40 AIS patients at 7 days after therapy. AIS, acute ischemic stroke.

To investigate the diagnostic power of miR-424 for acute ischemic stroke, ROC curves were constructed to compare the relative concentrations of miR-424 in lymphocytes and neutrophils in acute stroke patients and controls, and AUC was subsequently calculated. Generally, AUC>0.5 is considered diagnostic, whereas AUC>0.7, 0.7≤AUC≤0.9, and AUC>0.9 indicates a low, moderate and high diagnostic value, respectively. The AUC of miR-424 in lymphocytes is 0.652, indicating it indeed has diagnostic power for stroke, with a cut-off point of 2.696 to differentiate stroke patientsfrom controls with a sensitivity of 0.600 and a specificity of 0.900 (Fig. 1D, Table 2). The AUC of miR-424 in neutrophils is 0.637, indicating it also has diagnostic power for stroke, with a cut-off point of 1.300 to differentiate stroke patients from controls with a sensitivity of 0.600 and a specificity of 0.733 (Fig. 1D, Table 2). Hence, miR-424 levels in lymphocytes and neutrophils both have diagnostic value for ischemic stroke within 6 hours.

**Figure 3.**
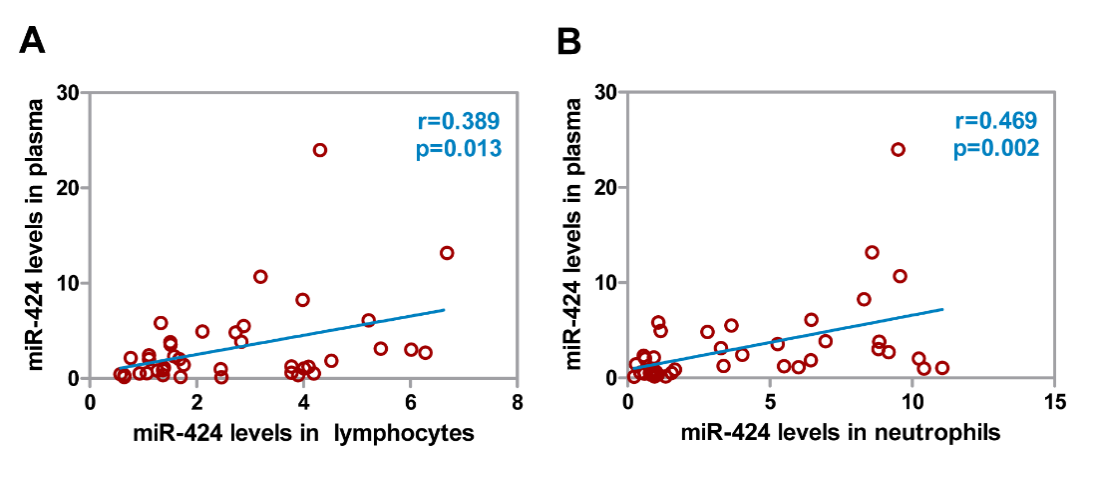
Correlations between miR-424 levels in circulating immune cells and in plasma. (A) Correlation between plasma miR-424 levels and its levels in lymphocytes from AIS patients. (B) Correlation between plasma miR-424 levels and its levels in neutrophils from AIS patients. AIS, acute ischemic stroke. N=40.

**Figure 4.**
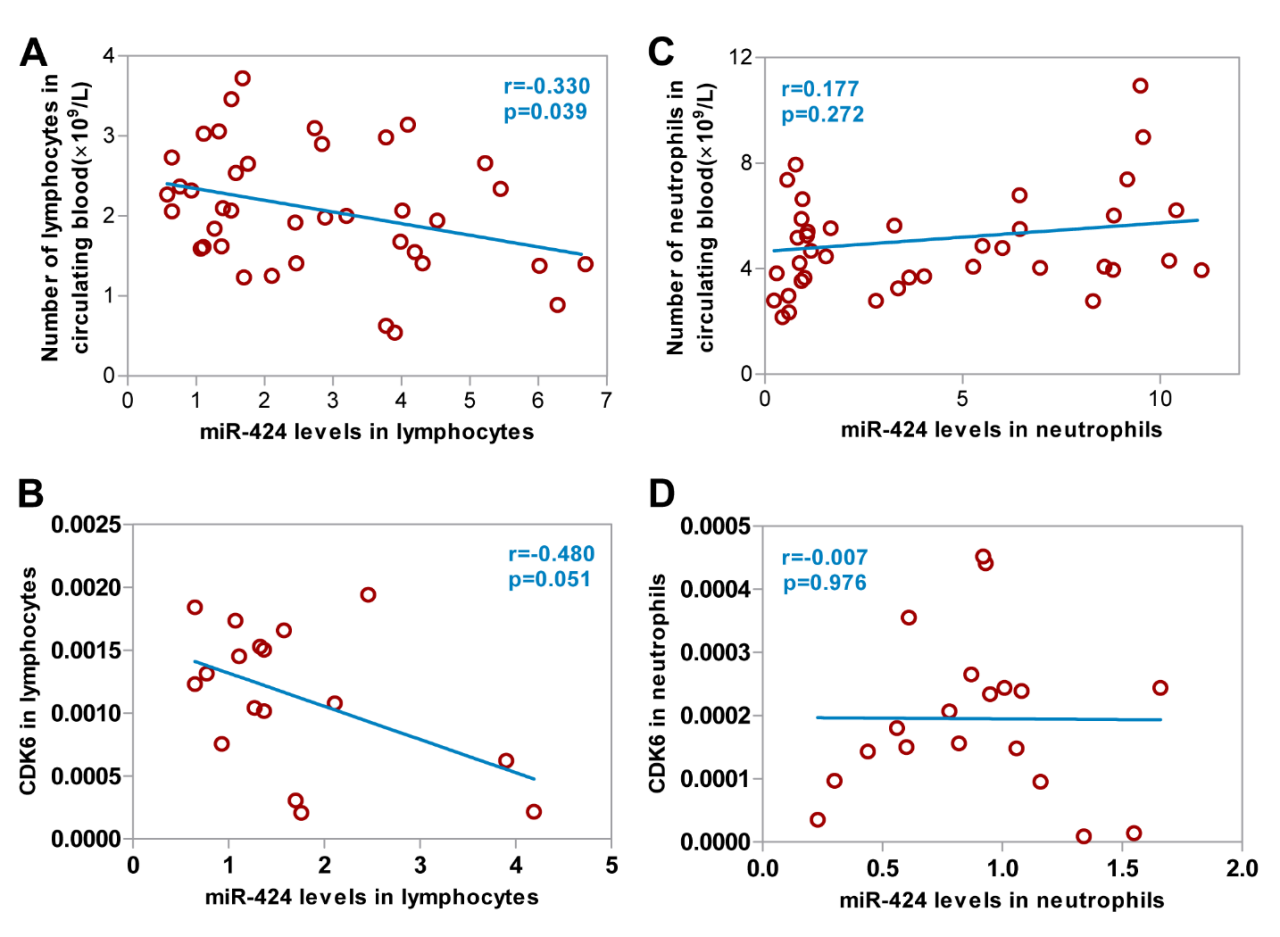
Correlations between miR-424 levels in lymphocytes and neutrophils, and the number of lymphocytes and neutrophils and CDK6 expression. (A) Correlation between miR-424 levels in lymphocytes and the number of lymphocytes from 40 AIS patients; (B) Correlation between miR-424 and CDK6 levels in lymphocytes from 19 AIS patients; (C) Correlation between miR-424 levels in neutrophils and the number of neutrophils from 40 AIS patients; (D) Correlation between miR-424 and CDK6 levels in neutrophils from 19 AIS patients. AIS, acute ischemic stroke.

### Negative correlation was found between miR-424 levels and infarct volume but not with neurological function score in AIS patients

We previously demonstrated the positive correlation between plasma miR-424 levels and Barthel Index, suggesting that it might be related to stroke severity. To assess the role of miR-424 in stroke severity, we examined the correlation between miR-424 levels and brain infarct volume, and a negative linear correlation was found between miR-424 levels in neutrophils and brain infarct volume within 72 hours of stroke onset (Fig. 2A, *p*<0.05). In order to further test whether miR-424 levels in lymphocytes or neutrophils were related to a neurological function score, we analyzed its correlation with NIHSS score at admission and at 7 days after thrombolytic therapy of AIS patients. However, we again did not find any linear correlation between miR-424 levels and NIHSS score (Fig. 2B-C).

### Correlations between miR-424 levels in lymphocytes or neutrophils and in plasma

Furthermore, correlation analysis showed that plasma miR-424 levels were positively correlated with expression in lymphocytes and neutrophils (Fig. 3A-B, *p*<0.05), indicating that plasma miR-424 might originate from lymphocytes and neutrophils. As was discussed in the aformentioned analysis, we believe that miR-424 in lymphocytes and neutrophils play important roles in the hyperacute stage of stroke.

**Figure 5.**
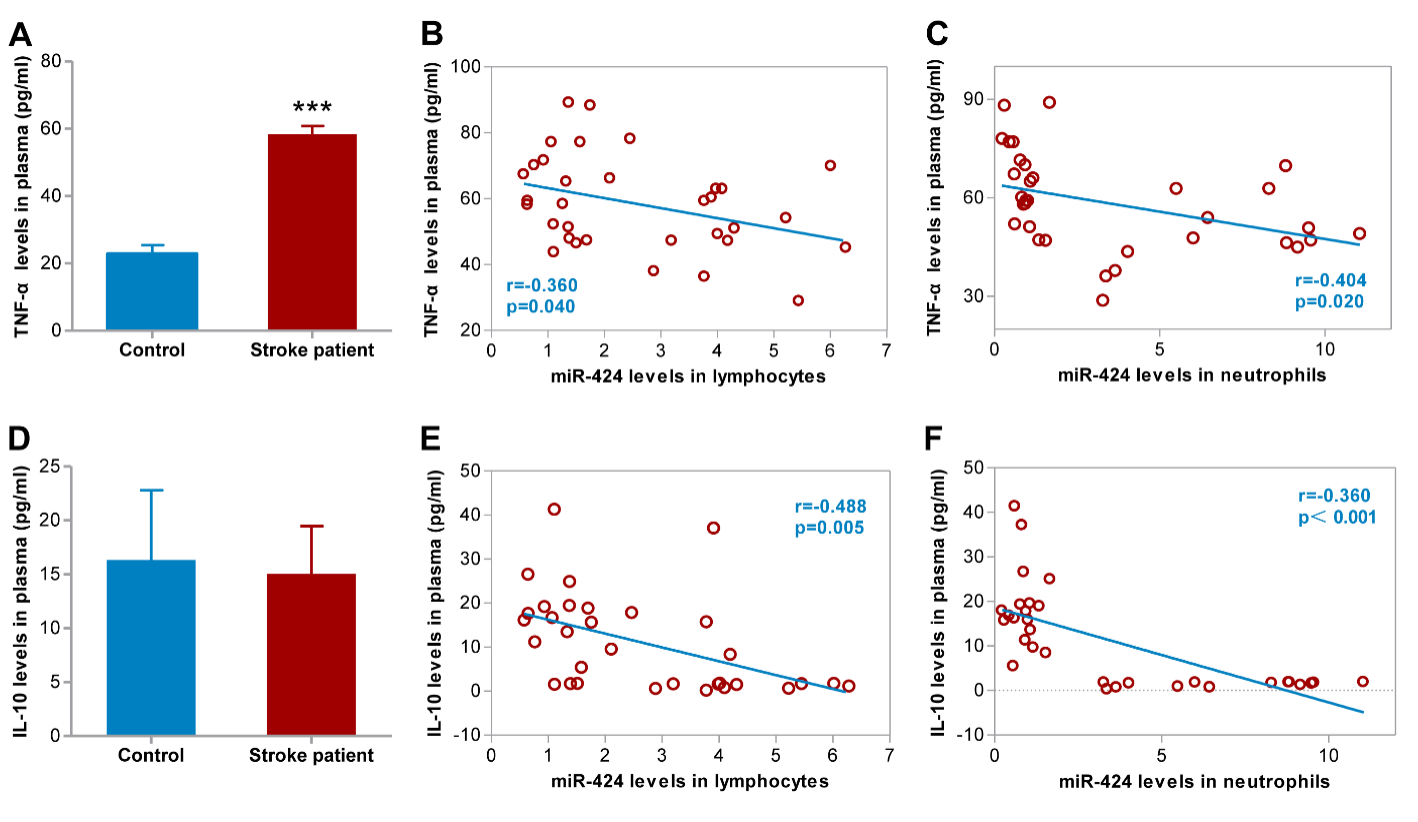
Correlations between changes in plasma TNF-α and IL-10 levels with miR-424 levels in lymphocytes and neutrophils. (A) TNF-α levels in plasma from 33 stroke patients and 24 control volunteers; ***p<0.001 compared to control. (B) Correlation between miR-424 levels in lymphocyte and TNF-α levels in plasma from 33 AIS patients. (C) Correlation between miR-424 levels in neutrophils and TNF-α levels in plasma from 33 AIS patients; (D) IL-10 levels in plasma from 33 stroke patients and 24 control volunteers. (E) Correlation between miR-424 levels in lymphocytes and IL-10 levels in plasma from 33 AIS patients; (F) Correlation between miR-424 levels in neutrophils and IL-10 levels in plasma from 33 AIS patients. Data represent mean±SEM. TNF-α, tumor necrosis factor-alpha; IL-10, interleukin-10. AIS, acute ischemic stroke.

### Lymphocytic miR-424 levels were negatively correlated with the number of lymphocytes and CDK6 levels

Our in vitro study demonstrated that overexpression of miR-424 resulted in a G1 phase cell-cycle arrest through a downregulation of cell cycle proteins CDK6 in microglia [12]. In order to assess the potential role of miR-424 in the proliferation of peripheral immune cells, we examined the correlation between miR-424 levels and the number of lymphocytes and neutrophils. We found that the number of lymphocytes decreased when miR-424 levels in lymphocytes of ischemic stroke patients within 6 hours increased (Fig. 4A, *p*<0.05). Furthermore, a negative correlation existed between lymphocytic miR-424 levels and CDK6 levels in lymphocytes (Fig. 4B). No linear correlation was found between miR-424 levels in neutrophils and the number of neutrophils (Fig. 4C), or between miR-424 levels and CDK6 levels in neutrophils (Fig. 4D).

### Correlations between miR-424 levels and plasma levels of TNF-α, IL-10, and IGF1

Inflammatory mechanisms in the CNS are essential in the pathophysiologic processes post-ischemic stroke [17, 18]. Given the anti-inflammatory role of miR-424 in the brain of experimental stroke, we further examined TNF-α, IL-10 and IGF1 levels in plasma and determined whether an association existed between plasma cytokines and miR-424 levels. We discovered that TNF-α and IGF1 levels in plasma dramatically increased and decreased, respectively, within 6 hours (Fig. 5A, 6A, *p*<0.05), whereas no significant changes in IL-10 were observed (Fig. 5D). Lymphocytic miR-424 levels are negatively correlated with TNF-α, IL-10 and IGF1 in plasma (Fig. 5E, 5F, 6B, *p*<0.05). A negative correlation between miR-424 levels in neutrophils and TNF-α, IL-10 and IGF1 in plasma was also confirmed (Fig. 5C, 5F, 6C, *p*<0.05).

**Figure 6.**
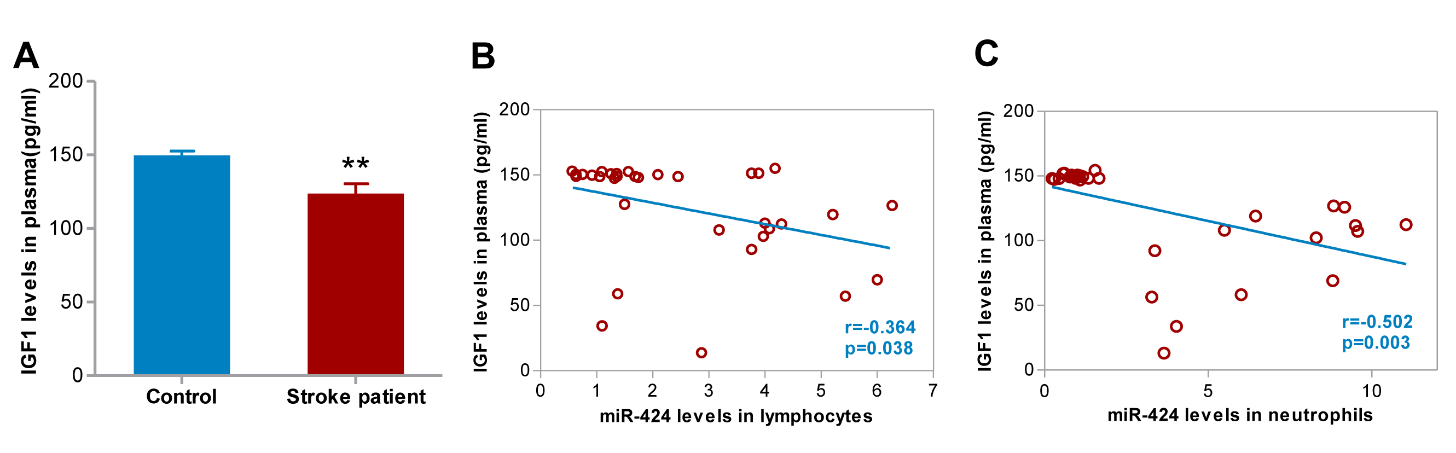
Correlations between change in plasma IGF1 levels with miR-424 levels in lymphocytes and neutrophils. (A) IGF1 levels in plasma from 33 stroke patients and 24 control volunteers; **p<0.01 compared to control. (B) Correlation between miR-424 levels in lymphocytes and IGF1 levels in plasma from 33 AIS patients; (C) Correlation between miR-424 levels in neutrophils and IGF1 levels in plasma from 33 AIS patients. IGF1(insulin-like growth factor 1),

## DISCUSSION

Previous studies have suggested that expression of various miRs in circulating blood is rapidly altered after stroke onset, and some specific miRs have the potential to be ischemic stroke biomarkers. This is the first report to investigate the clinical value of miR-424 in peripheral immune cells including lymphocytes, neutrophils and plasma from the acute ischemic stroke patients. We found that elevated miR-424 levels in peripheral immune cells has potential as a diagnostic tool for ischemic stroke, and may have an immunosuppressive effect through inhibiting lymphocyte proliferation by targeting CDK6, resulting in less release of cytokines and neurotrophic factor from lymphocytes and neutrophils, which subsequently affects the severity of acute cerebral infarction.

First, to determine the diagnostic and prognostic potential of miR-424 for acute ischemic stroke, we detected its changes, as well as its correlation, with neurological function deficit score and cerebral infarct volume. Here we found that miR-424 levels in lymphocytes and neutrophils are markedly upregulated within 6 hours of stroke onset. Moreover, high levels of miR-424 in neutrophils was correlated with lower cerebral infarct volume; however, elevated miR-424 levels were not related to neurological function deficits at admission or at 7 days following treatment. Next, we attempted to analyze whether plasma miR-424 originates from lymphocytes and neutrophils of stroke patients. Correlation analysis showed that plasma miR-424 levels were positively correlated with expression in both lymphocytes and neutrophils, suggesting that plasma miR-424 may leak from both kinds of peripheral immune cells. From the aforementioned analysis, we believe miR-424 in lymphocytes and neutrophils play important roles in the diagnostic and pathologic processes of the hyperacute stage of stroke.

The immune response after stroke plays a major role in ischemic brain. The peripheral and central immune cells can significantly predict and affect the clinical outcome of stroke. Thus, they constitute a potential target for therapeutic approaches and stroke prevention. We previously showed that exogenous overexpression of miR-424 in the brain protects against permanent focal cerebral ischemia injury via suppression of microglia-mediated inflammatory response by reducing the expression of cell cycle proteins CDK6, cyclin D1, CDC25A [12]. To further study whether miR-424 levels in peripheral immune cells might influence the pathological process of stroke, we analyzed the correlations between miR-424 levels and the numbers of lymphocytes and neutrophils, as well as the plasma cytokines levels in ischemic stroke. In the present study, negative correlations were found between lymphocytic miR-424 levels and the number of lymphocytes, as well as CDK6 levels, indicating that the elevated lymphocytic miR-424 levels might depress lymphocyte proliferation and the expression of CDK6. Moreover, we found that pro-inflammatory cytokine TNF-α level in plasma increased and neurotrophic factor IGF-1 [19-22] in plasma decreased after stroke, both of which were negatively correlated with miR-424 levels in lymphocytes or neutrophils. Taken together, the above results indicate that the upregulated miR-424 levels in circulating immune cells might depress the proliferation of lymphocyte, as well as reduce the levels of pro-inflammatory cytokine (TNF-α), anti-inflammatory cytokine (IL-10), and neurotrophic factor (IGF-1) in plasma, demonstrating the immunosuppressive potential of miR-424 in peripheral immune response for AIS patients.

In summary, we provide the first evidence of the diagnostic and immunosuppressive potential of miR-424 in circulating immune cells for AIS patients. Our investigation showed that miR-424 was not only involved in the inhibition of the inflammatory response of central nervous system, but also in the depression of the peripheral inflammatory response through CDK6-dependent pathway in lymphocytes, or CKD6-independent pathway in neutrophil. However, given the immunosuppressive potential of miR-424, in addition to a negative correlation between miR-424 levels and cerebral infarct volume, we still could not speculate whether the upregulation of miR-424 in peripheral immune cells was beneficial or detrimental for ischemic stroke patients. Additional experimental investigations, including a systematic study with a large sample size to further evaluate the clinical significance of miR-424 for stroke treatment are necessary.
